# Chlorella virus pyrimidine dimer glycosylase and *Escherichia coli* endonucleases IV and V have incision activity on 2,2,4-triamino-5(2*H*)-oxazolone

**DOI:** 10.1186/s41021-015-0022-8

**Published:** 2015-11-01

**Authors:** Katsuhito Kino, Masayo Suzuki, Masayuki Morikawa, Takanobu Kobayashi, Shigenori Iwai, Hiroshi Miyazawa

**Affiliations:** Kagawa School of Pharmaceutical Sciences, Tokushima Bunri University, 1314-1 Shido, Sanuki, Kagawa 769-2193 Japan; Division of Chemistry, Graduate School of Engineering Science, Osaka University, Osaka, Japan

**Keywords:** Guanine oxidation, Oxazolone, Chlorella virus pyrimidine dimer glycosylase, *Escherichia coli* endonucleases IV, *Escherichia coli* endonucleases V

## Abstract

**Introduction:**

2,2,4-Triamino-5(2*H*)-oxazolone (Oz) in a DNA strand is an oxidation product of guanine and 8-oxo-7, 8-dihydroguanine, and such a lesion can cause G-to-C transversions. Previously, Fpg/Nei and Nth were shown to have incision activity on Oz.

**Findings:**

We investigated the activities of chlorella virus pyrimidine dimer glycosylase (cvPDG) and *Escherichia coli* endonucleases IV (Nfo) and V (Nfi) on Oz. Although the three enzymes have different repair mechanisms from Fpg/Nei and Nth, they still had incision activity on Oz.

**Conclusions:**

Given the incision activities of cvPDG, Nfo and Nfi on Oz in addition to Fpg/Nei and Nth, Oz is DNA damage that can be repaired by diverse enzymes.

## Introduction

**Fig. 1:**
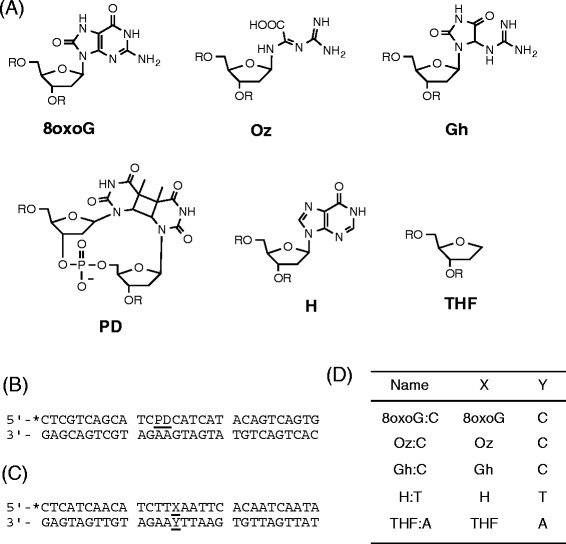
Structures of DNA damages and sequences containing DNA damages. **a** Structures of guanine oxidation products, cyclobutane thymine dimer (PD), hypoxanthine (H) and tetrahydrofuran (THF). **b** The sequence of the DNA duplex containing PD. The top strand contained PD and was labeled with ^32^P at the 5′ end (indicated by *). **c** The sequences of the DNA substrates. The top strand contained a lesion at position X and was labeled with ^32^P at the 5′ end (indicated by *). **d** X and Y in the nucleotides represent each lesion

Endogenous and exogenous oxidative stress causes DNA damage, and several enzymes repair this damage [1, 2]. Among the four bases, guanine is most susceptible to oxidative damage. Although 8-oxo-7,8-dihydroguanine (8oxoG) (Fig. 1a) is known to be a guanine oxidation product and a typical oxidation marker, 8oxoG has lower oxidation potential than guanine and is more readily oxidized. Thus, 8oxoG can be further oxidized, and oxidation products other than 8oxoG would be expected to have biological effects. 2,2,4-Triamino-5(2*H*)-oxazolone (Oz) is produced from guanine and 8oxoG by several oxidants: one-electron transfer [3]; superoxide radical [4]; singlet oxygen [5]; hydroxyl radical [6]; γ-radiation [3]; peroxynitrite [7]; and iodine [8]. Two to six molecules of Oz per 10^7^ guanines have been detected in liver DNA [9]. Although *Escherichia coli* DNA polymerase inserted adenine opposite Oz and Oz caused G-to-T transversions in *E. coli* cells [10, 11], we recently reported that eukaryotic DNA polymerases α, β, δ, and ε almost exclusively inserted guanine opposite Oz [12, 13] and incorporation of adenine was dependent on families of DNA polymerases [12]. Importantly, cytosine can be incorporated opposite 8oxoG, but not opposite Oz. Therefore, Oz is a pre-mutagenic lesion that can cause G-to-C or G-to-T transversions in eukaryotes, and so repair of Oz is required to prevent point mutations.

Previously, *E. coli* Fpg and Nth enzymes were shown to excise Oz from dsDNA oligomers with similar efficiencies regardless of the type of base in the opposite strand [10, 14]. Recently, we found that human NEIL1 (hNEIL1) and NTH1 (hNTH1) can excise Oz [15]. Herein, we report the incision efficiency of chlorella virus pyrimidine dimer glycosylase (cvPDG) and *E. coli* endonucleases IV (Nfo) and V (Nfi) on Oz.

## Materials and methods

### Materials

Oligonucleotides containing a single Oz, guanidinohydantoin (Gh) and cyclobutane thymine dimer (PD) were prepared as described previously [10, 16–18]. The oligonucleotides containing a single 8oxoG, hypoxanthine (H) and tetrahydrofuran (THF) were purchased from Nihon BioService. Nfo and Nfi were purchased from New England Biolabs. cvPDG was purchased from Trevigen.

### PAGE analysis of nicking reactions with enzymes

The oligonucleotide (30mer) containing Oz, Gh, PD, hypoxanthine, 8oxoG and THF was 5′ end-labeled by treatment with T4 polynucleotide kinase and [γ-^32^P]ATP and purified. The sequences of the oligomers used are shown in Fig. 1b-d. The nicking reactions (5 μl) were performed in mixtures containing the following components: (for Nfo) 50 mM Tris-HCl, pH 7.9, 100 mM NaCl, 10 mM MgCl_2_, 1 mM DTT, and 0.5 μg BSA, (for Nfi) 20 mM Tris-acetate, pH 7.9, 50 mM potassium acetate, 10 mM magnesium acetate, 1 mM DTT, and 0.5 μg BSA, (for cvPDG) 25 mM sodium phosphate, pH 6.8, 100 mM NaCl, 1 mM EDTA, 1 mM DTT, and 1 μg BSA. The ^32^P-labeled DNA (100 fmol), complementary oligomer (200 fmol) and each enzyme were incubated at 30 °C for 1 h. Reactions with enzymes were stopped by adding an equal volume of dye solution containing EDTA, heated at 70 °C for 5 min, and subjected to 16 % denaturing PAGE. Radioactivity was quantified using the BAS2500 bioimaging analyzer (Fujifilm).

## Results and discussion

### The incision of oligonucleotides containing Oz by pyrimidine dimer glycosylase

**Fig. 2:**
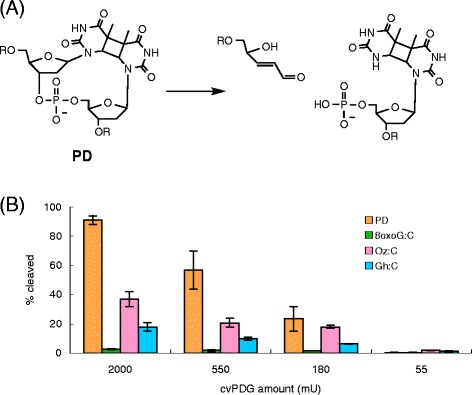
Incision activities of chlorella virus pyrimidine dimer glycosylase (cvPDG). **a** The scheme of the cleaved oligonucleotides containing PD (Fig. 1) using cvPDG [19, 20]. **b** The percentage of the cleaved oligonucleotides in the ^32^P-labeled DNA substrate with varied amounts of cvPDG was plotted as a graph. The mean values and standard errors were calculated from at least two independent experiments

cvPDG cleaves glycosidic bonds of the 5′-pyrimidine of a cyclobutane pyrimidine dimer, followed by cleavage of phosphodiester bonds (Fig. 2a) [19, 20]. Moreover, 8oxoG is not a substrate of cvPDG, but formamidopyrimidines [21]. Thus, we investigated the incision activities of cvPDG on Oz-containing duplex DNA, which were compared with those on PD as the positive control lesion (Fig. 2b). Figure 2b shows that Oz is able to be cleaved by cvPDG, but the observed activities on Oz are lower than those on PD. Thus, in addition to formamidopyrimidines [21], Oz is also a substrate of cvPDG.

Gh is an oxidation product of 8oxoG, and DNA polymerases incorporate adenine and guanine opposite Gh [16]. Since the behavior of Gh in DNA replication somewhat resembles that of Oz, we compared the activity on Gh with Oz. Figure 2b shows that cvPDG incises Oz more efficiently than Gh, and much more than 8oxoG. It was previously reported that the order of piperidine reactivity is Oz > Gh > 8oxoG, and the order of the *N*-glycosidic bond strength is Oz < Gh < 8oxoG [15]. When DNA glycosylases do not recognize a specific lesion, cleavage of glycosidic bonds by DNA glycosylases is related to the *N*-glycosidic bond strength [15]. Thus, moderate repair efficiency of Oz by cvPDG seems to be due to weak *N*-glycosidic bond strength at Oz rather than accurate recognition of Oz by cvPDG.

### The incision of oligonucleotides containing Oz by endonuclease IV (Nfo)

**Fig. 3:**
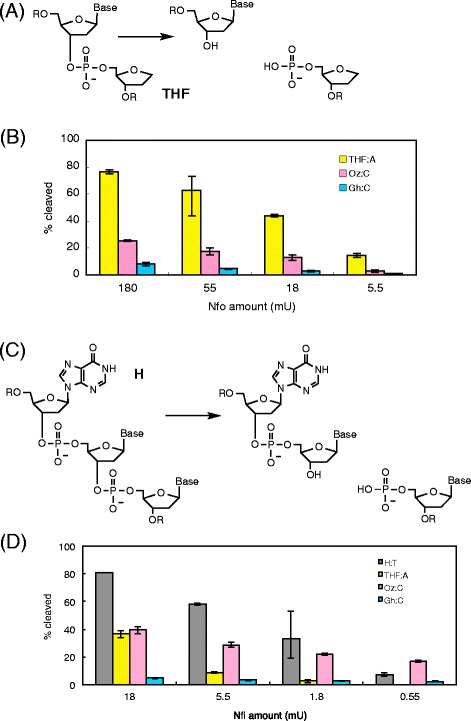
Incision activities of *E. coli* endonuclease IV (Nfo) and endonuclease V (Nfi). **a**,**c** The scheme of the cleaved oligonucleotides using Nfo [19, 21] (**a**) and Nfi [19, 22] (**c**). **b**,**d** The percentage of the cleaved oligonucleotides in the ^32^P-labeled DNA substrate with varied amounts of Nfo (**b**) and Nfi (**d**) was plotted as a graph. The mean values and standard errors were calculated from at least two independent experiments

Nfo is an apurinic/apyrimidinic endonuclease, and it hydrolyzes the phosphodiester bond 5′ to an abasic site (Fig. 3a) [19, 22]. The catalytic mechanism of Nfo is quite different from that of Nei, Nth, hNEIL1 or hNTH1. We investigated the incision activities of Nfo on the Oz-containing duplex DNA and compared them with those on THF (Fig. 1a) as a stable mimic of the abasic site. The results in Fig. 3b indicate that the observed activity of Nfo on Oz is one-third to one-fourth of that on THF. Thus, Nfo can moderately repair Oz.

In Fig. 3b, Nfo incised Oz more efficiently than Gh. Since Gh has a five-membered ring structure, Gh is less similar to the abasic site than Oz (Fig. 1a). Thus, it seems that Nfo repairs Oz more efficiently than Gh due to its similarity to the abasic site. Taken together, Nfo appears to be better suited for being a backup repair enzyme for Oz, than for Gh.

### The incision of oligonucleotides containing Oz by endonuclease V (Nfi)

**Fig. 4:**
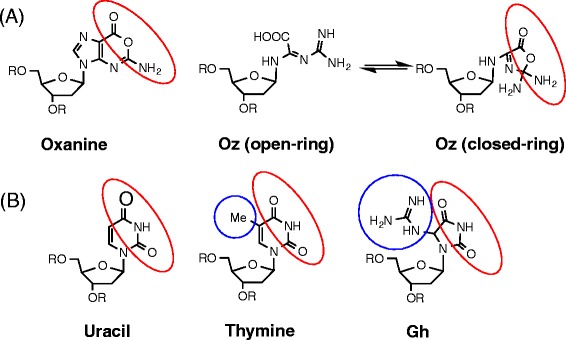
Structures of DNA lesions and possible recognition site of Nfi. **a** Structures of oxanine and the possible open- or closed-ring structure of Oz. Red circle indicates the common moiety that Nfi may recognize. **b** Structures of uracil, thymine and Gh. Red circle indicates the common moiety that Nfi may recognize. Blue circle indicates the moiety protruding from the six- or five-membered ring, and Nfi may disfavor these moieties

Nfi is a deoxyinosine 3′ endonuclease. Nfi cleaves at the second phosphodiester bond 3′ to the hypoxanthine residue (Fig. 3c) [19, 23], and the catalytic mechanism of Nfi is quite different from that of Nei, Nth, hNEIL1 or hNTH1. It is possible that Nfi recognizes a wide variety of substrates [23]. Therefore, we investigated the incision activity of Nfi on the Oz-containing duplex DNA, which were compared with that on hypoxanthine (Fig. 1a) as the positive control lesion. In addition, the incision activity of Nfi at the Oz lesion was compared with that at THF. The results in Fig. 3d indicate that the observed activity on Oz is lower than that on hypoxanthine at high concentrations of Nfi, and is higher than that on THF. Since THF has no base moiety, Nfi does not readily recognize THF, and these data indicate that Oz has some recognition sites for Nfi. Since Nfi recognizes oxanine (Fig. 4a) in DNA [23] and Oz is possible to have the open- or closed-ring structure (Fig. 4a), the closed-ring structure of Oz similar to oxanine (Fig. 4a) is thought to be able to react with Nfi.

It was shown that Nfi was able to recognize Oz much more efficiently than Gh (Fig. 3d). Previously, Nfi was shown to recognize uracil but not thymine in DNA, suggesting that the 5′-methyl group is critical for recognition by Nfi (Fig. 4b) [23]. Gh has a moiety protruding from the ring as does thymine (Fig. 4b), thus Nfi may have low incision activity against oligonucleotides containing Gh. Nfi is therefore better suited for being a backup repair enzyme for Oz, than for Gh.

### Implications and conclusion

We described our analysis of incision reactivities on Oz with various repair enzymes. Human 8-oxoguanine DNA N-glycosylase 1 and human apurinic/apyrimidinic endonuclease 1 have no activity on Oz [15, 24], thus these enzymes are not repair enzymes for Oz. In contrast, hNEIL1 and hNTH1 enzymes have high reactivities on Oz, even though these enzymes erroneously incise at Oz sites of Oz:G and Oz:A [15]. The results in this paper reveal that Nfo, Nfi and cvPDG also have moderate activities on Oz compared with each positive control lesion. Nonetheless, these three enzymes incised Oz more efficiently than Gh, thus they may serve as a backup for repair of Oz.

## Abbreviations

cvPDG: Chlorella virus pyrimidine dimer glycosylase
Nfo: *E. coli* endonuclease IV
Nfi: *E. coli* endonuclease V
hNEIL1: human NEIL1
hNTH1: human NTH1
Oz: 2,2,4-Triamino-5(2*H*)-oxazolone
8oxoG: 8-oxo-7,8-dihydroguanine
Gh: guanidinohydantoin
PD: cyclobutane thymine dimmer
H: hypoxanthine
THF: tetrahydrofuran
